# Central Myxoma / Myxofibroma of the Jaws: A Clinico-Epidemiologic Review

**Published:** 2017-01

**Authors:** Agbara Rowland, Fomete Benjamin, Obiadazie Athanasius-Chukwudi, Omeje Uchenna-Kevin, Samaila Modupeola-Omotara

**Affiliations:** 1 *Department of Oral and Maxillofacial Surgery, Jos University Teaching Hospital, Jos, Plateau state, Nigeria.*; 2 *Department of Oral and Maxillofacial Surgery, Ahmadu Bello University Teaching Hospital, Shika-Zaria, Kaduna state, Nigeria.*; 3 *Department of Oral and Maxillofacial Surgery, Aminu Kano University Teaching Hospital, Kano, Nigeria. *; 4 *Department of Pathology/Morbid Anatomy, Ahmadu Bello University Teaching Hospital, Shika-Zaria, Kaduna state, Nigeria.*

**Keywords:** Jaw, Odontogenic Myxoma, Myxofibroma

## Abstract

**Introduction:**

Myxomas are a group of benign rare tumors of connective-tissue origin that occur in both hard (central) and soft tissues of the body. The aim of this study is to highlight our experience in the management of central myxoma of the jaw, with emphasis on its clinic-epidemiologic features as seen in our environment.

**Materials and Methods:**

All patients who were managed for central myxoma of the jaw at the Oral and Maxillofacial Surgery department of a regional University Teaching Hospital between September 1997 and October 2015 were retrospectively studied. Details sourced included age, sex, site of tumor, duration, signs/symptoms, treatment given, and complications. Data were analyzed using Statistical Package for Social Sciences (SPSS) version 16 (SPSS Inc., Chicago, IL, USA) and Microsoft Excel 2007 (Microsoft, Redmond, WA, USA). Results from descriptive statistics were represented in the form of tables and charts, with a test for significance (*ρ)* using Pearson Chi-square (χ^2^) set at 0.05.

**Results:**

A total of 16 patients were managed within the period reviewed, consisting of 10 (62.5%) females and six (37.5%) males, giving a male-to-female ratio of 1:1.7. The ages of patients ranged from 5 to 70 years, with a mean of 27.06±15.45 years. The mandible accounted for nine (56.3%) cases and the maxilla for six (37.5%) cases, while a combination of the maxilla and the zygoma were involved in one (6.3%) case. Bucco-lingual or bucco-palatal expansion were the most common presentation (six [46.2%] cases each). Histological assessment of tissue specimens showed that fibromyxoma accounted for seven (43.8%) cases, while the remaining nine (56.3%) cases were diagnosed as myxoma. All patients had jaw resections, and these consisted of mandibulectomies in nine (60.0%) patients and maxillectomies in six (40.0%) patients. The duration of hospital stay ranged from 5 to 29 days, with a mean of 17.86±7.68 days. Complications were noted in three patients, and all were surgical wound infections.

**Conclusion:**

Most patients in our environment present late with large tumors and are usually not compliant with follow-up review. Thus, a radical approach is favored in most patients.

## Introduction

Myxomas are a group of benign rare tumors of connective-tissue origin that occur in both hard (central) and soft tissues of the body (1-4). Although benign in nature, they are locally aggressive and tend to recur if poorly treated. In general, the left ventricle of the heart is the most common site of occurrence (5). Soft tissue myxomas have been classified into intramuscular myxoma, juxta-articular myxoma, superficial angiomyxoma, aggressive angiomyxoma and neurothekeoma (nervous sheath myxoma), and each type shows predilection to certain regions of the body and to either gender (6). These tumors have been reported in sites such as the thigh, shoulder, gluteal region, upper limb, pelvis and perineum, skin and subcutaneous tissues (4,7,8). The human jaw is the most common site affected in skeletal (central) involvement. Other reported sites include the clavicle, femur, and calcaneum (4,9). The criterion for the diagnosis of myxoma (the presence of fusiform and stellate cells embedded in a pulpy mucoid stroma lacking chondroblast, lipoblast, striated muscle, and other differentiated cells with few tumor vessels and monocentric orientation) was established by Stout (10).

In the head and neck region, central myxomas have been reported (outside the jaw) in the skull base, nasal bone, temporal bone, sphenoidal sinus, palate, axis and zygoma (11-14). The cheek, gingiva, larynx, parotid, eyelid, conjunctiva, external auditory canal and the nasal vestibule are some of the documented sites of involvement in head and neck soft tissue myxoma (15).

Although the World Health Organization (WHO) classified myxoma of the jaws under odontogenic tumors, the exact histogenesis of jaw myxomas is not fully understood, and both odontogenic and nonodontogenic primitive tissues have been suggested as precursors of tumor cells (16,17).

The words myxoma and fibromyxoma are commonly used interchangeably. Although fibromyxomas contain larger collagen bundles compared with myxomas, there appears to be no difference in their biologic behavior (18). Myxomas may exist as an isolated tumor or as part of a syndrome such as Mazabraud’s syndrome (a combination of one or more intramuscular myxomas and fibrous dysplasia of bone) or Carney’s syndrome (characterized by skin pigmentary abnormalities, myxomas, endocrine tumors or overactivity, and schwannomas) (19,20). Odontogenic myxoma is the second most common odontogenic jaw tumor (after ameloblastoma) in sub-Saharan Africa, accounting for between 12% and 16% of odontogenic tumors (21). Generally, the incidence of myxoma of the jaw varies from one region to another and is more commonly reported in females than males (3,17,22). Myxomas have a greater predilection for the mandible than the maxilla and affect all age groups, although individuals between the second and fourth decade of life appear to be more predisposed (23,24). The clinical presentation of jaw myxomas depends on the site of the tumor (lower or upper jaw; posterior or anterior location) and extent of jaw involvement among other factors. This retrospective study highlights our findings in the management of 16 cases.

## Materials and Methods

All patients who presented at the Oral and Maxillofacial Surgery clinic of a regional University Teaching Hospital between September 1997 and October 2015 with tumor of the jaws which was histologically diagnosed as myxoma/myxofibroma were retrospectively studied. Information was sourced from patient case notes and operating theater records. Details sourced included age, sex, site of tumor, duration, signs/symptoms, treatment given, and complications. Data retrieved were analyzed using Statistical Package for Social Sciences (SPSS) version 16 (SPSS Inc., Chicago, IL, USA) and Microsoft Office Excel 2007 (Microsoft, Redmond, WA, USA). Findings from descriptive statistics are presented in the form of graphs, tables and charts. The test for statistical significance (*p)* using Pearson Chi-square (χ^2^) was set at P<0.05.

## Results

**Fig 1:**
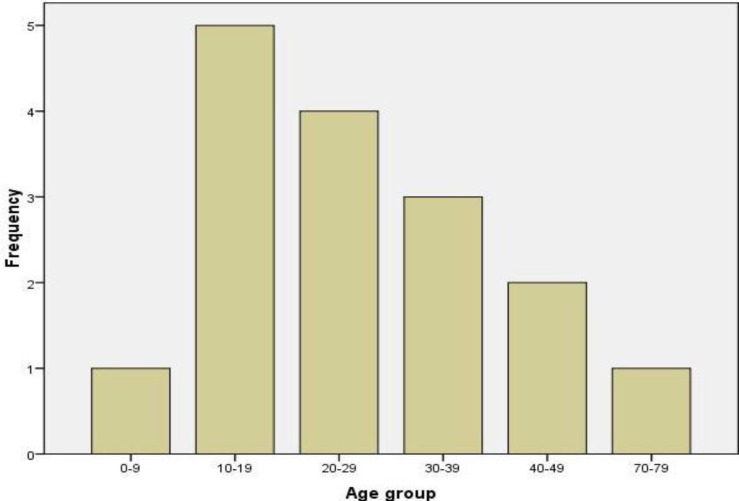
Age range of patients

*Age and sex: *A total of 16 patients were managed within the period reviewed, consisting of 10 (62.5%) females and six (37.5%) males, giving a male-to-female ratio of 1:1.7. The ages of patients ranged from 5 to 70 years, with a mean age of 27.06±15.45 years. The highest incidence was recorded in patients within the second to fourth decade of life (Fig.1).

*Clinical presentation, radiological assessment and histology*

**Fig 2:**
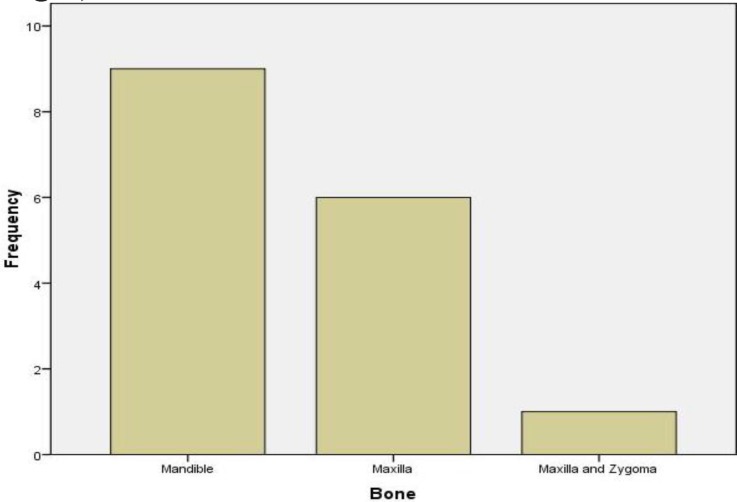
Facial bones involved in myxoma

The main presenting complaint in all patients was jaw swelling, with a duration that ranged from 3 months to 168 months (mean, 42.4 months). Other presentations were tooth mobility in five (31.25%) patients and oral ulceration in one (6.3%) patient. With reference to the site of jaw swelling, the mandible accounted for nine (56.25%) cases and the maxilla for six (37.5%) cases, while a combination of the maxilla and zygoma were involved in one (6.3%) case (Fig.2). 

The left side of the mandible was involved in four (26.7%) cases, while the left side of the maxilla was involved in three (20.0%) cases. The right sides of the jaws were involved in three (20.0%) cases each. Bilateral mandibular involvement was noted in two (13.3%) cases.

The pattern of cortical involvement was documented in 13 of the 16 patients studied and these were bucco-lingual in six (46.2%) cases, bucco-palatal in six (46.2%) cases, and buccal in one (7.7%) case. There was no statistically significant relationship between duration of swelling and pattern of cortical involvement (P=0.100). The premolar–molar region was involved in four patients, while the anterior-molar region was involved in three patients. There was no documentation of the extent in the remaining nine patients.

All patients were reviewed using only plain radiographs. However, detailed radiological findings were missing from the records.

Histological assessment of tissue specimens showed that fibromyxoma accounted for seven (43.8%) cases, while the remaining nine (56.3%) cases were diagnosed as myxoma. There was no statistically significant relationship between the ages of the patients and the histological variant (P=0.696). Similarly, there was no statistically significant relationship between the sex of patients and histological type (P=0.084).

*Treatment and complications*

Treatment modality used in patient management was documented in 15 of the 16 patients. All patients had jaw resections and these consisted of partial mandibulectomies in nine (60.0%) patients and partial maxillectomies in 6(40.0%) patients (Table.1). One patient required elective tracheostomy due to difficult intubation. None of the patients had reconstruction post-jaw resection.

The duration of hospital stay ranged from 5 to 29 days, with a mean of 17.86±7.68 days. Complications were noted in three patients and all were surgical wound infections.

**Table 1:** Type of resection used

**Resection type**	**Frequency**	**Percent**
Limited maxillectomy	3	20.0
Hemimaxillectomy	2	13.3
Marginal resection	2	13.3
Segmental mandibulectomy	1	6.7
Subtotal mandibulectomy	1	6.7
Segmental mandibulectomy with disarticulation	4	26.7
Subtotal mandibulectomy with disarticulation	2	13.3
Total	15	100.0
		

***Follow-up***

The postoperative period of follow-up ranged from 6 weeks to 14 months. No recurrence was recorded within this period of follow-up.

## Discussion

The higher female-to-male ratio in central myxomas of the jaws recorded in this study is similar to some previous findings (23,25). However, some other studies recorded either a higher female-to-male ratios (17,26) or an equal sex distribution. (27,28)

The reason for our observed increased female predilection in both the mandible and maxilla, and in all races, is not known. However, it may be due to genetic factors.

In this study, the highest incidence of central jaw myxoma was recorded in the 10–19-year age group followed by the 20–29- and 30–39-year age groups, similar to other studies (23,26,29). Only a single case was observed in the elderly. Generally, central myxoma of the jaws is rare in children and the elderly, and is essentially a tumor of the young. Four of the patients in this study were in the pediatric age group, with the youngest being 5 years of age. Other studies have reported occurrence in the pediatric population below 5 years of age (23,30). Although rare in these age groups, central myxoma should always be considered in the differential diagnosis of common lesions of the jaw bearing similar clinical and radiological features in order to avoid inappropriate treatment.

The mandible accounted for a slightly higher number of cases (56.3%) when compared with the maxilla, and this pattern has been reported in previous studies (24,26,31). However, equal incidences in the mandible and maxilla have also been reported (29,32).

The clinical presentation in the central myxoma of the jaws depends among other factors on the site and extent of the disease. An obvious jaw swelling was the most common clinical presentation noted in this study. Other findings were mobile tooth, toothache (preceding jaw swelling), and mucosal ulceration. Previous studies documented a slow growing jaw swelling as the main clinical presentation (5,29,31), and this is usually painless except when associated with infection or neural affectation. Mucosal ulceration is not a frequent finding even in large tumors. Only a single case in our study presented with frank mucosal ulceration. Factors responsible for mucosal ulceration noted in our environment included trauma from opposing teeth (with subsequent superimposed infection), incision using hot knives by traditional health practitioners, and the use of corrosive traditional mouth rinses. With regard to cortical involvement, central myxoma of the jaws usually involves both cortices (bucco-lingual or bucco-palatal expansion). Fifteen of the 16 patients in this study presented with bicortical bony expansion. The premolar-molar region accounted for the highest location of myxomas and this finding is in agreement with previous reports (23). Despite the massive proportion of jaw myxomas seen in this environment, they rarely cross the midline. In this study, only two cases crossed the midline, and this is in agreement with other studies (23,29).

The definitive diagnosis of myxoma is histological; however, radiological examination assists in surgical planning, with plain (conventional) radiography being the imaging modality commonly used. Advanced imaging techniques such as computed tomography (CT) and magnetic resonance imaging (MRI) may assist in overcoming some of the limitations associated with plain radiography. Conventional radiological findings in the central myxoma of the jaws has been classified into types I–VI based on appearance of the lesion, and into well-defined, poorly defined and diffuse, based on the definition of the lesion border (33). Findings in the central myxoma of the jaw on plain radiograph are those of a radiolucent lesion which may be unilocular clear, unilocular with trabeculations, or multilocular in appearance, and this is commonly associated with tooth displacement. The lesion may not be corticated, or it may be poorly corticated, moderately corticated, or well corticated (34). Other causes of unilocular or multilocular radiologic appearance such as ameloblastoma, keratocystic odontogenic tumor, calcifying epithelial odontogenic tumor, fibro-osseous lesions, and central giant cell granuloma should be considered in the differential diagnosis of myxoma of the jaws. Tooth root resorption is not a frequent finding, although a high incidence has been reported (25). CT findings are those of a hypodense or isodense lesion. Other information possible with CT includes degree of cortication, presence of locularity, growth pattern (lobular, budding or crevices), and extension into the surrounding structures. CT scan provides better information on the degree of cortication and expansion when compared with plain radiographs (34,35). MRI differentiates between the myxoid and collagenous component of the tumor by their signal intensities (collagenous part enhances of T1 weighted images while the myxomatous component does not enhance or weakly enhances), and precisely differentiates between tumor tissue and normal soft tissue. However, tooth displacement is poorly demonstrated on both CT and MRI scans (35). Plain radiography was the only imaging modality used in this study. In the absence of a CT or MRI scan, a combination of extraoral and intraoral views in resource-depleted environments provides reasonable information that is useful for surgical planning. The lack of an advanced imaging technique in this study is due to its non-availability in our center prior to 2005 and an inability of patients to afford the cost.

**Fig 3:**
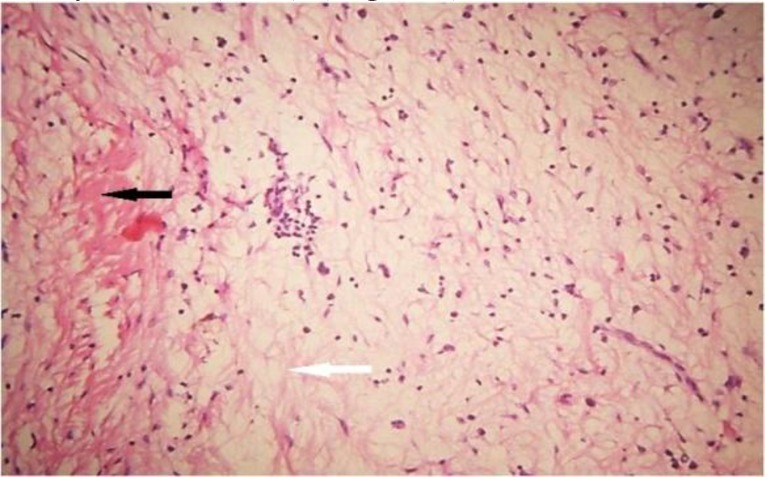
Photomicrograph of fibromyxoma showing proliferation of spindle and stellate cells in a myxoid stroma (white arrow) with fibrous tissue (black arrow). H&E ×200

**Fig 4:**
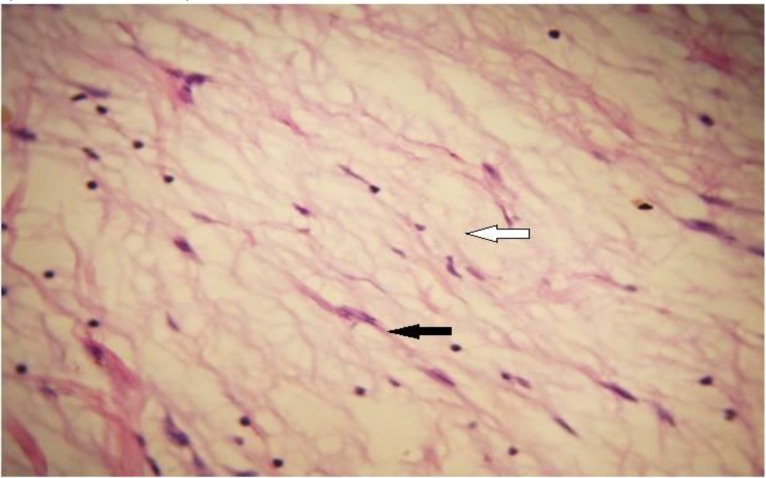
Photomicrograph shows proliferation of spindle and stellate cells (black arrow) in a myxoid stroma (white arrow). H&E ×400

Histologically, myxoma is largely an unencapsulated tumor composed of spindled or stellate-shaped cells in a mucoid-rich intercellular matrix composed of glycosaminoglycan and rich in hyaluronic acid with mitotic figures and multinucleated cells rarely found (4,29) (Figs 3,4). 

The stroma contain a small amount of collagen fibrils; however, some cases contain a larger amount of collagen bundles and are thus referred to as myxofibroma or fibromyxoma. Myxofibroma accounted for 43.7% of cases in this study, and this is higher than other reported values (31,36). Positive staining of the mucoid-rich extracellular matrix for Alcian blue and Periodic acid-Schiff have been well reported. Tumor cells show uniform positivity on immunostaining with vimentin and patchy staining with smooth muscle actin, although they were negative for desmin, neuron-specific enolase, glial fibrillary acid protein, neurofilaments and S100 (29,36). Myxoid-containing lesions should be considered in the histologic differential diagnosis of myxoma, and these include pleomorphic adenoma, nodular fascitis, myxoid lipoma, fibrous dysplasia, chondromyxoid fibroma, myxoid neurofibroma, myxofibrosarcoma, rhabdomyosarcoma, and liposarcoma.

**Fig 5:**
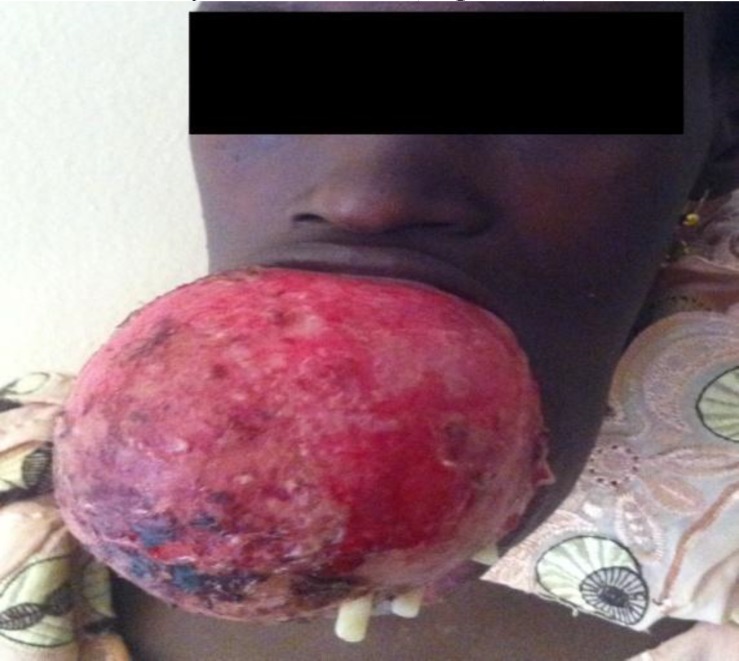
Massive central myxoma of the jaws

**Fig 6:**
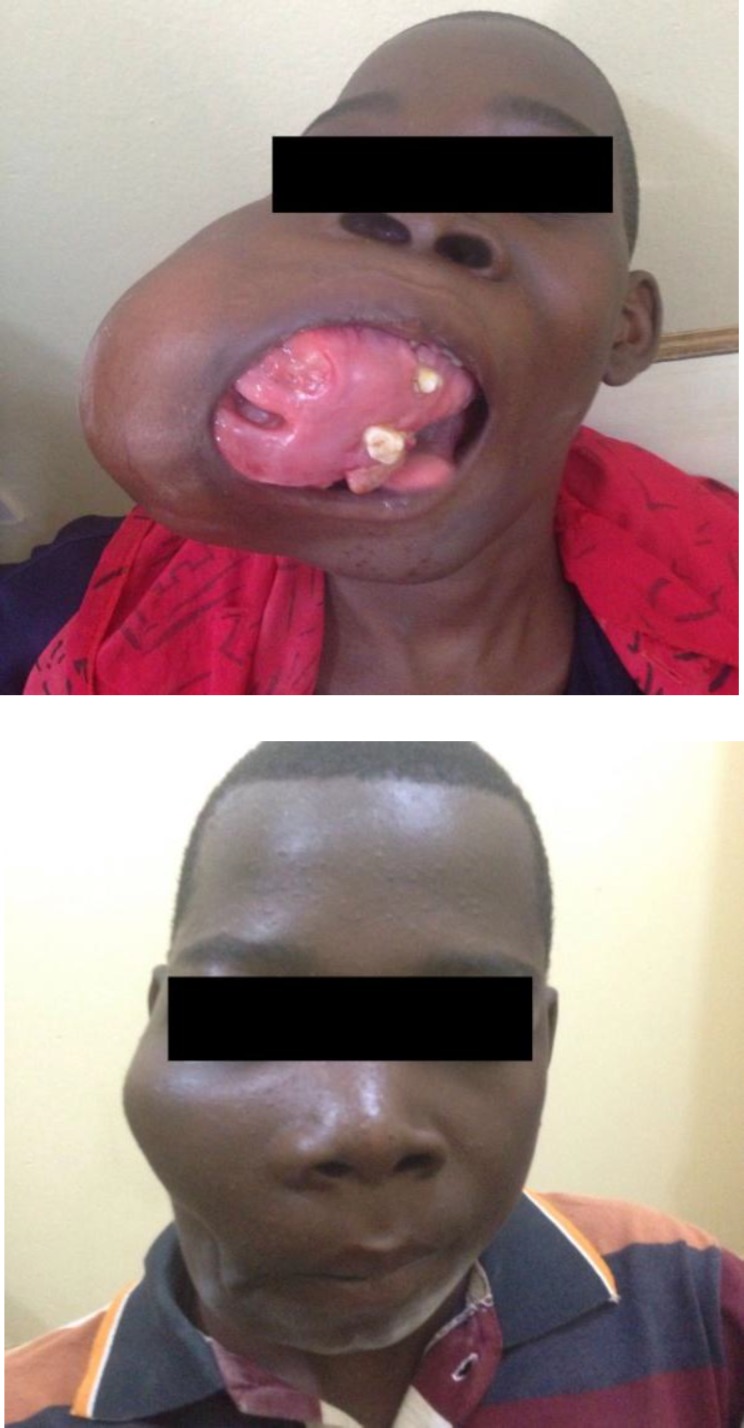
Central myxoma of the jaw in a child. (a) Preoperative view. (b) Postoperative appearance following jaw resection

The treatment of myxoma is surgical, and different surgical methods such as curettage, enucleation, and resection have been advocated. However, conservative methods (curettage and enucleation) have been associated with a higher recurrence rate (3,24). All patients studied in this retrospective analysis were treated using a radical surgical approach (mandibulectomies and maxillectomies) (Table.1). Important considerations in the choice of treatment include site and extent of tumor, patient level of education, motivation for follow-up, and whether or not the tumor is recurrent. Most patients in our environment present late with large tumors and are financially constrained (Figs 5,6). 

In addition, compliance with follow-up review is typically poor; thus, a radical approach is favored in most patients. The margin of resection is usually based on radiological assessment plus additional consideration of tumor behavior. Once the margins have been decided radiologically, odontogenic myxoma is further treated surgically in the same manner as ameloblastoma (ie, with a margin of 1.5–2.0 cm from the radiological margin). However, the periosteum should be preserved where feasible, especially in younger patients. Preservation of the periosteum has been associated with spontaneous bone regeneration as reported by one of the authors (37). None of the patients treated had reconstruction post jaw resection, and this is mainly due to financial reasons and the absence of advanced reconstruction techniques such as microvascular surgery, especially in partial maxillectomy cases.

Follow-up of patients postoperatively was poor, and only five of the 16 patients managed were available for follow-up and this was for a short period of time. The period of follow-up ranged from 1 month to 14 months and no recurrence was noted. However, this period may be inadequate to assess effectiveness of treatment, although recurrence following treatment of myxoma has been reported as early as 3 months and as late as 15 yrs (4,38). It is our opinion that follow-up of patients should be for life where feasible. Periodic serial radiographs should also be taken during the follow-up period to detect early recurrence.

## Conclusion:

Most patients in our environment present late with large tumors and are usually not compliant with follow-up review. Thus, a radical approach is favored in most patients.
